# Suppression of proline-directed protein kinase F_A_expression inhibits the growth of human chronic myeloid leukaemia cells

**DOI:** 10.1054/bjoc.1999.1133

**Published:** 2000-03-21

**Authors:** C-P Hsu, S-F Hsueh, C-C Yang, S-D Yang

**Affiliations:** Department of Life Science, National Tsing Hua University, Hsinchu, 30013, Taiwan, Republic of China

**Keywords:** chronic myeloid leukaemia cells K562, PDPK F_A_, cell growth

## Abstract

Initial studies revealed that proline-directed protein kinase F_A_(PDPK F_A_) was overexpressed in various cancerous tissues relative to normal controls. However, the functional role of overexpressed PDPK F_A_in cancer remains to be established. In this report, we explore the potential role of PDPK F_A_in leukaemia cell growth by investigating the effects of partial inhibition of this kinase on the malignant phenotype of human chronic myeloid leukaemia cells (K562). Cloning of PDPK F_A_cDNA and its recombinant antisense expression vector and PDPK F_A_-specific antibody were successfully developed. Two stable antisense clones of K562 cells were subcloned which expressed 70% and 45% of PDPK F_A_respectively, compared with control-transfected clone in both immunoprecipitate activity assay and immunoblot analysis. In sharp contrast, these two antisense clones expressed no significant suppression of any other related PDPK family members, indicating the specificity of these two antisense clones. Moreover, these antisense clones proportionally and potentially exhibited cell growth retardation, poor clonogenic growth in soft agar and loss of serum independence. The results demonstrate that specific antisense suppression of PDPK F_A_is sufficient to interfere with the growth of K562 cells, indicating that PDPK F_A_is essential for human chronic myeloid leukaemia cell growth. © 2000 Cancer Research Campaign


British Journal of Cancer (2000) **82**(8), 1480–1484
© 2000 Cancer Research Campaign
DOI: 10.1054/ bjoc.1999.1133, available online at http://www.idealibrary.com on IDEAL®

### Suppression of proline-directed protein kinase $F_A$ expression inhibits the growth of human chronic myeloid leukaemia cells

**C-P Hsu, S-F Hsueh, C-C Yang and S-D Yang**

Department of Life Science, National Tsing Hua University, Hsinchu 30013, Taiwan, Republic of China

**Summary** Initial studies revealed that proline-directed protein kinase $F_A$ (PDPK $F_A$) was overexpressed in various cancerous tissues relative to normal controls. However, the functional role of overexpressed PDPK $F_A$ in cancer remains to be established. In this report, we explore the potential role of PDPK $F_A$ in leukaemia cell growth by investigating the effects of partial inhibition of this kinase on the malignant phenotype of human chronic myeloid leukaemia cells (K562). Cloning of PDPK $F_A$ cDNA and its recombinant antisense expression vector and PDPK $F_A$-specific antibody were successfully developed. Two stable antisense clones of K562 cells were subcloned which expressed 70% and 45% of PDPK $F_A$ respectively, compared with control-transfected clone in both immunoprecipitate activity assay and immunoblot analysis. In sharp contrast, these two antisense clones expressed no significant suppression of any other related PDPK family members, indicating the specificity of these two antisense clones. Moreover, these antisense clones proportionally and potentially exhibited cell growth retardation, poor clonogenic growth in soft agar and loss of serum independence. The results demonstrate that specific antisense suppression of PDPK $F_A$ is sufficient to interfere with the growth of K562 cells, indicating that PDPK $F_A$ is essential for human chronic myeloid leukaemia cell growth.
© 2000 Cancer Research Campaign

**Keywords:** chronic myeloid leukaemia cells K562; PDPK $F_A$; cell growth

Proline-directed protein kinase $F_A$ (PDPK $F_A$) was originally identified as type-1 protein phosphatase activating factor/glycogen synthase kinase-3α (Vandenheede et al, 1980; Hemmings et al, 1981; Woodgett, 1990), but has subsequently been demonstrated as a multisubstrate PDPK possibly involved in the regulation of diverse cell functions (for reviews, see Plyte et al, 1992; Yang, 1994). Initial clinical studies revealed that PDPK $F_A$ was over-expressed many-fold in various human cancerous tissues relative to normal controls and the expression of PDPK $F_A$ activity appeared to be significantly correlated with the progression states of various human cancers (Lee et al, 1995; Yang et al, 1995, 1996, 1998). This provided an initial evidence that overexpression of PDPK $F_A$ may be involved in tumour promotion and progression. However, the exact functional role of overexpressed PDPK $F_A$ in cancer has not yet been directly demonstrated and remains to be established.

In this report, we use a more direct approach to investigate the potential role of PDPK $F_A$ in controlling the growth of K562 cells which were developed from leukaemia cells of a chronic myeloid leukemia blastic phase patient by Lozzio and Lozzio (1975). We have successfully cloned a partial sequence of PDPK $F_A$ cDNA and constructed a recombinant antisense expression vector. The antisense expression vector could be stably transfected into K562 cells. This results in a specific suppression of PDPK $F_A$ expression, and concomitantly leads to cell growth retardation, decreased serum independence and poor clonogenic growth in soft agar. The results presented here demonstrate that specific antisense suppression of PDPK $F_A$ expression is sufficient to interfere with the growth properties of K562 cells, indicating that PDPK $F_A$ is essential for human chronic myeloid leukaemia cell growth. Suppression of overexpressed PDPK $F_A$ may provide a useful clinic target for therapeutic intervention aimed at inhibiting the malignant behaviour of human chronic myeloid leukaemia.

*Received 9 September 1999*
*Revised 1 December 1999*
*Accepted 3 December 1999*

*Correspondence to:* S-D Yang

### MATERIALS AND METHODS

### Materials

Human chronic myeloid leukaemia cells (K562), derived from leukaemia cells of a chronic myeloid leukaemia blastic phase patient, were supplied by American Type Culture Collection (ATCC, Rockville, MD, USA). The cells within passages 10–30 were used for all the experiments in this text. [γ-$^{32}$P]ATP and enhanced chemiluminescence (ECL) Western blotting detection reagents were purchased from Amersham (Buckinghamshire, UK). RPMI-1640, fetal bovine serum and geneticin (G418) were from Gibco (Paisley, UK). Benzamidine, n-α-tosyl-L-lysine chloromethyl ketone (TLCK), phenylmethylsulphonyl fluoride (PMSF), sodium fluoride (NaF), Triton X-100, LiCl and Tween-20 were from Merck (Darmstadt, Germany). 3-(4,5-dimethylthiazal-2-yl)-2,5-diphenyltetrazolium bromide (MTT), sodium orthovanadate and 3-(cyclohexylamino)-1-propane-sulphonic acid (CAPS) were from Sigma (St Louis, MO, USA). BCA (bacteriochlorine *a*) protein assay reagent was from Pierce (Rockford, IL, USA). Protein A-Sepharose CL-4B was from Pharmacia (Uppsala, Sweden). Polyvinylidene fluoride (PVDF) membrane (Immobilon-P) was from Millipore (Bedford, MA, USA).

Phospho GS-2, mouse monoclonal anti-GSK-3β and rabbit polyclonal anti-Erk-1/-2 antibodies were from UBI (Lake Placid, NY, USA). Goat anti-rabbit and anti-mouse IgG antibodies conjugated with peroxidase were from Bio-Rad (Hercules, CA, USA). Aprotinin and DOTAP transfection kit were from Boehringer Mannheim (Mannheim, Germany). X-ray film was from Fuji (Tokyo, Japan).

### Production of anti-PDPK $F_A$ antibody

The peptide QAPDATPTLTNSS, corresponding to the carboxyl terminal region from amino acids 471–483 of the sequence of $F_A$ (Woodgett, 1990) was synthesized by peptide synthesizer (model 9050, Milligen, Bedford, MA, USA). The cysteine residue was added to the $NH_2$ terminus in order to facilitate coupling of the peptide to bovine serum albumin according to the procedure described by Reichlin (1980) using glutaraldehyde as the cross-linker. The detailed procedure for production and affinity-purification of this antibody was as described in previous reports (Yu and Yang, 1993, 1994; Yang et al, 1998).

### Cloning of PDPK $F_A$ cDNA and construction of recombinant antisense expression vector

A partial sequence (~1.0 kb fragment starting from 3′ end of $F_A$ cDNA) was cloned from human fibroblast cells by reverse transcriptase polymerization chain reaction using 5′-CGCGGCCT-GGAAGAGGCCAG-3′, 5′-ACTGGAGGTGGGGACAGGGA-3′, 5′-AAGCTAGCGCCTGTGCTCGGCGCCATGA-3′ and 5′-TTG-AATTCGCCCTCAGGAGGAGTTAGTG-3′, as primers (Woodgett, 1990; He et al, 1995). The cloned cDNA fragment was constructed into pBK-CMV vector in an antisense orientation downstream of the cytomegalovirus (CMV) promoter using *Eco*RI–*Nhe*I as the ligation sites. The neomycin-resistant gene placed downstream of the sinian virus 40 origin was used as the second open reading frame for initial screening of the transfected clones. The developed antisense construct named as At$F_A$pBK-CMV was put into mass production in *Escherichia coli* and plasmid was purified by alkaline lysis method.

### Cell culture and selection of stably-transfected clones

K562 cells were cultured in RPMI-1640 medium supplemented with 10% heat-inactivated fetal bovine serum, 25 U ml$^{-1}$ penicillin and 25 μg ml$^{-1}$ streptomycin. Cells were incubated at 37°C in a 95% air/5% carbon dioxide ($CO_2$) and water-saturated atmosphere in 75-cm$^2$ flasks. For transfection, pBK-CMV vector alone as control, or At$F_A$pBK-CMV vector as antisense construct as described above was introduced into cells by DOTAP. Briefly, 10 μg vector mixed with DOTAP was incubated with $1 \times 10^6$ cells in serum-free medium at 37°C for 6 h. The transfected cells were then seeded in 96-well plate ($1 \times 10^4$ cells per well) with complete medium containing 400 μg ml$^{-1}$ geneticin (G418) for selection of recombinant clones expressing G418 resistance. After 4 weeks, individual clones surviving in the presence of G418 were further expanded to mass culture.

### Cell extract preparation

For cell extract preparation, $2.5 \times 10^6$ cells washed twice with ice-cold phosphate-buffered saline (PBS) were suspended in 500 μl solution A (10 mM Tris–HCl at pH 7.4, 2 mM EDTA, 1 mM EGTA, 50 mM sodium chloride (NaCl), 1% Triton X-100, 1 mM PMSF, 1 mM benzamidine, 0.5 μg ml$^{-1}$ aprotinin, 50 mM NaF, 20 mM sodium pyrophosphate and 1 mM sodium orthovanadate) and then homogenized on ice by Sonic Dismembrator (model 150, Fisher) for 3 × 10 s at 40% power output. Cell lysates were then ultracentrifuged at 100 000 ***g*** for 30 min at 4°C and the supernatants were used as the cell extracts. Protein concentration of the cell extract was determined by BCA protein assay and adjusted to 1 mg ml$^{-1}$ with solution A.

### Immunoprecipitation and PDPK $F_A$ activity assay in the immunoprecipitate

Cell extract (300 μg cell protein) was incubated with 0.5 μl affinity-purified anti-PDPK $F_A$ antibody (1.5 μg pure IgG) at 4°C for 1.5 h and then with 100 μl protein A-Sepharose CL-4B (20% vv in solution A) for another 1.5 h with shaking. The immunoprecipitate was collected by centrifugation, washed twice with solution B (20 mM Tris–HCl at pH 7.0, 0.5 mM dithiothreitol (DTT), 1 mM PMSF, 1 mM benzamidine, and 0.1 μM aprotinin) containing 0.5 M LiCl, once with 1 ml solution B, and then resuspended in 200 μl solution B. For PDPK $F_A$ activity assay in the immunoprecipitate, 10 μl immunoprecipitate at appropriate dilution were incubated with 15 μl mixture containing 20 mM Tris–HCl at pH 7.0, 0.5 mM DTT, 10 μg heparin, 0.2 mM [γ-$^{32}$P]ATP (~1000 cpm pmol$^{-1}$), 20 mM magnesium chloride ($MgCl_2$) and 60 μM phospho GS-2 (YRRAAVPPSPSLSRHSSPHQ-**p**SEDEEE) at 30°C for 10 min. $^{32}$P-incorporation was measured by spotting 20 μl reaction mixture on phosphocellulose paper (1 × 3 cm$^2$), washing 3 times with 75 mM phosphoric acid ($H_3PO_4$) and counting in liquid scintillation counter. A unit of PDPK $F_A$ is that amount of enzyme that incorporates 1 pmol of phosphate per min into the peptide substrate.

### Immunoblot analysis

For immunoblot analysis, the cell extract containing 10 μg cell protein was subjected to 10% sodium dodecyl sulphate polyacrylamide gel electrophoresis (SDS-PAGE), electrotransferred to PVDF membrane and then immunoblotted with 50 ng ml$^{-1}$ primary antibodies as indicated and then with goat anti-rabbit or anti-mouse IgG antibody conjugated with peroxidase (1:3000) essentially as described in previous reports (Yang et al, 1994, 1998; Yu and Yang, 1994). Immunoblot was developed with the ECL system using peroxidase substrate at 25°C for chemiluminescence detection (Gillespie and Hudspeth, 1991). The luminescent light emission was recorded on X-ray film and quantified by computing densitometer (Molecular Dynamics, Sunnyvale, CA, USA).

### Clonogenic growth in soft agar

Antisense and control-transfected clones of K562 cells were plated in soft agar for clonogenicity as described by Jeha et al (1996). Briefly, a feeder layer consisting of medium supplemented with 20% serum and 0.5% agarose was plated in a 60-mm tissue culture dish. The feeder layer was overlaid with a top layer that included $1 \times 10^4$ cells with 20% serum and 0.35% agarose. After 10 days, colonies were stained with 2 ml of 1 mg ml$^{-1}$ MTT solution at 37°C for 12 h and then counted and photographed.

*© 2000 Cancer Research Campaign*

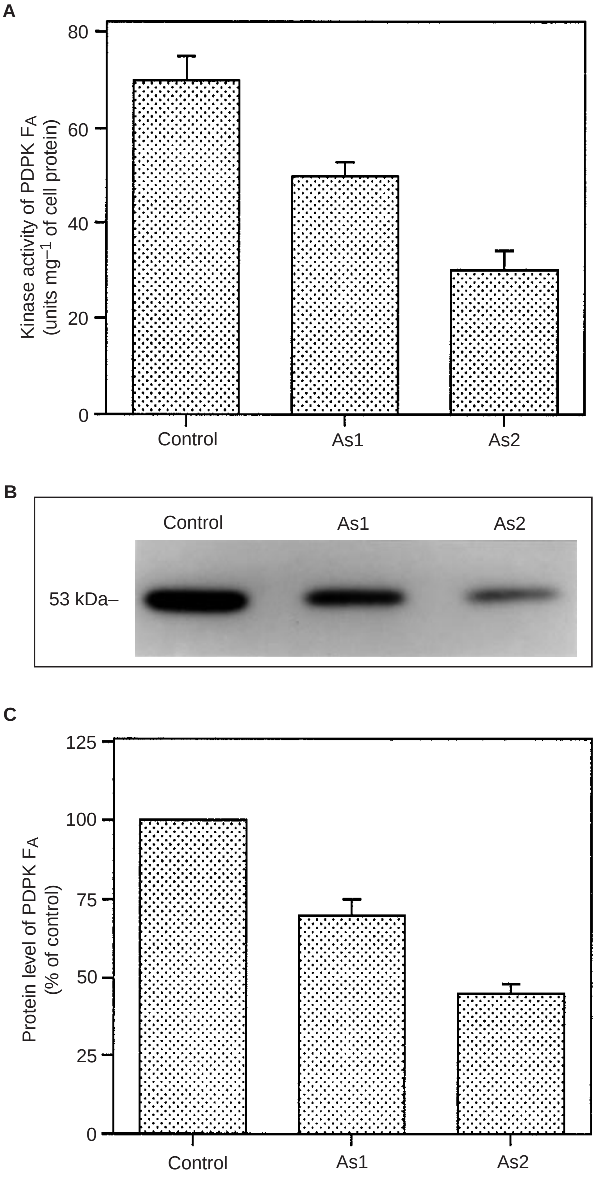

**Figure 1** Suppression of PDPK $F_A$ protein expression in antisense clones of K562 cells. The cell extracts (300 µg cell protein) of PDPK $F_A$ antisense (As1 and As2) and control-transfected (control) clones as indicated were immunoprecipitated by using 1.5 µg anti-PDPK $F_A$ antibody. The PDPK $F_A$ activity in the immunoprecipitate was measured using phospho GS-2 as substrate (**A**). The same cell extracts containing 10 µg cell protein were subjected to 10% SDS-PAGE, electrotransfered to PVDF membrane and immunoblotted with 50 ng $ml^{-1}$ of anti-PDPK $F_A$ antibody (**B**) followed by computing densitometric quantification of the relative amount of PDPK $F_A$ (% of control) on the immunoblot (**C**). Data were taken from the representative results of three independent experiments and expressed as means ± s.d. As1/As2: the two antisense clones of K562 cells stably transfected with PDPK $F_A$ antisense expression vector

**Table 1** Biological properties of PDPK $F_A$ antisense clones of K562 cells

| Cell clones[a] | Protein level of PDPK $F_A$[b] (% of control) | Doubling time[c] (H) | Soft agar colonies[d] (% Colonies/dish) |
|---|---|---|---|
| Control | 100 | 18.2 ± 0.6 | 100 |
| As1 | 70 | 20.1 ± 0.4 | 12.2 ± 0.5 |
| As2 | 45 | 24.3 ± 0.3 | 2.4 ± 0.8 |

[a]Control, control-transfected clone; As1/As2, the two PDPK $F_A$ antisense clones. [b]The relative amount of PDPK $F_A$ determined by immunoblot analysis. [c]Antisense or control clones ($2.5 \times 10^4$ cells $ml^{-1}$) were cultured at 37°C in medium supplemented with 10% serum and counted every 24 h by trypan blue exclusion method. The doubling time was calculated during the exponential growth phase. Results are the averages of four independent experiments and expressed as means ± s.d. [d]Antisense or control clones ($1 \times 10^4$ cells/dish) in a top layer consisting of medium supplemented with 20% serum and 0.35% agarose were overlaid on the feeder layer consisting of medium supplemented with 20% serum and 0.5% agarose in 60-mm culture dish. After 10 days at 37°C, colonies were stained with 2 ml $mg^{-1}$ $ml^{-1}$ MTT solution at 37°C for 12 h. Data were taken from the averages of three independent experiments and expressed as % of control.

### RESULTS

### Establishment and characterization of antisense clones that stably induced a decrease of PDPK $F_A$

Transfection of K562 cells was performed with $AtF_A$pBK-CMV vector as the antisense construct or with pBK-CMV vector as control following G418 selection as described in Materials and Methods. Several G418-resistant clones were successfully subcloned and expression levels of PDPK $F_A$ were determined by immunoprecipitate kinase assay for cellular activity and by immunoblot analysis for protein expression. Similar protein and activity levels (70 ± 5 units $mg^{-1}$ cell protein) of PDPK $F_A$ were found to exist in both untransfected parental and control-transfected clones (data not further illustrated). The results indicate that neither transfection nor G418 treatment could affect the expression of PDPK $F_A$ in K562 cells. In contrast, two antisense clones were obtained in which the cellular activities of PDPK $F_A$ were decreased to the levels of 50 ± 3 units $mg^{-1}$ cell protein (As1) and 30 ± 4 units $mg^{-1}$ cell protein (As2) respectively (Figure 1A). Immunoblot analysis revealed that the protein levels of PDPK $F_A$ in these two antisense clones were also suppressed in a similar manner (Figure 1B). Computing densitometric analysis further revealed that the protein levels of As1 and As2 were suppressed to 70% and 45% of control level respectively (Figure 1C). The suppressed activity (Figure 1A) and protein (Figure 1 B, C) levels of PDPK $F_A$ in the antisense clones appeared to be very similar, demonstrating that inhibition of PDPK $F_A$ activity in antisense clones is due to suppression of the protein expression. Moreover, although the DNA sequence of PDPK $F_A$ has 85% identity with glycogen synthase kinase-3β (GSK-3β) (Woodgett, 1990), the PDPK $F_A$ antisense expression vector constructed here appeared to have no significant effect on the protein expression of GSK-3β in K562 cells (not shown). We also determined another well-established PDPK member, namely the mitogen-activated protein kinases (MAPKs), and again no significant effect on the expression of Erk-1/-2 in these two antisense clones could be observed (not shown). The results demonstrate that the antisense expression vector constructed here specifically suppressed PDPK $F_A$ in K562 cells and had no effect on the expression of the other DNA sequence-homologous PDPK members such as GSK-3β and MAPKs.

### Suppression of PDPK $F_A$ expression in antisense clones resulted in blocking the growth of K562 cells

To study the role of PDPK $F_A$ in controlling the growth of K562 cells, the biological properties of antisense expressing K562 cells

 *© 2000 Cancer Research Campaign*

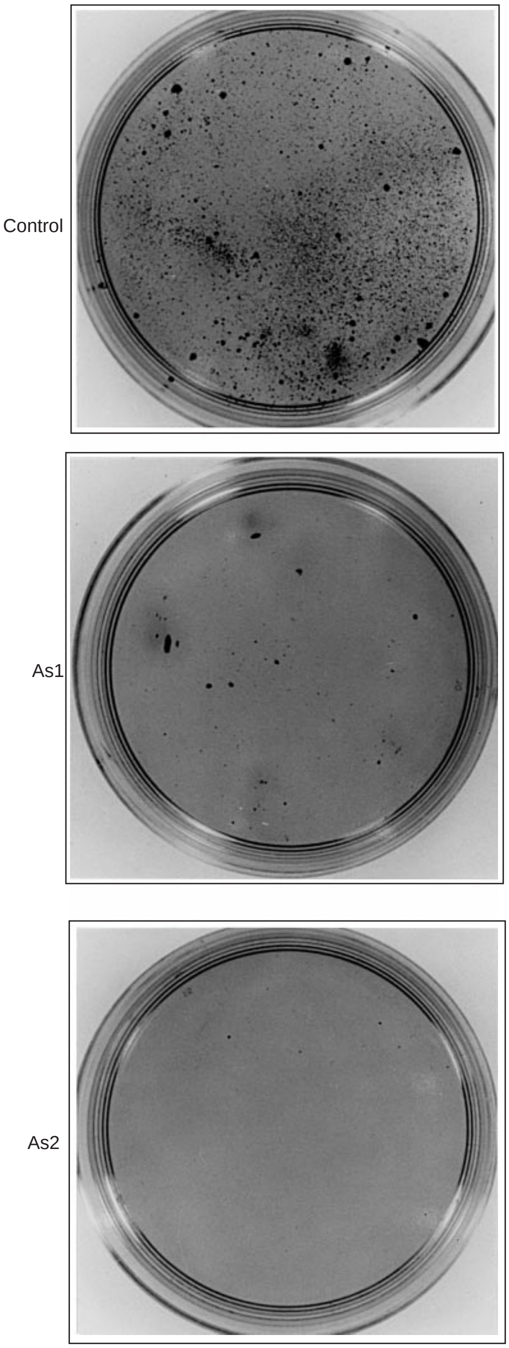

**Figure 2** Assessment of clonogenicity of PDPK $F_A$ antisense clones in soft agar. Clonogenicity of PDPK $F_A$ antisense (As1 and As2) and control-transfected (control) clones was assessed by growing $1 \times 10^4$ cells in 0.35% agarose to form colonies as described in Materials and Methods. After 10 days, the colonies stained with MTT solution at 37°C for 12 h were photographed. Data were taken from the representative results of three independent experiments

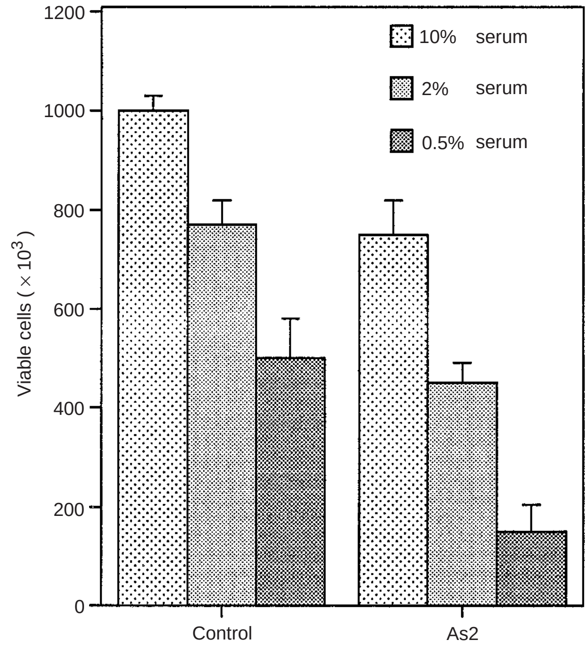

**Figure 3** Serum-dependent growth in PDPK $F_A$ antisense clone. PDPK $F_A$ antisense (As2) and control-transfected (control) clones ($2.5 \times 10^5$ cells) were cultured in different concentrations of serum as indicated at 37°C for four days and the viable cells were counted by trypan blue exclusion method. Results are the averages of four independent experiments and expressed as means ± s.d were examined. In studying the growth property of the antisense clones, we found that the two antisense clones proportionally had lower growth rates compared with control-transfected cells. Doubling time, calculated during the exponential growth phase, was significantly and proportionally increased from 18.2 h of control up to 24.3 h as summarized in Table 1. Since clonogenic growth in soft agar was established as an important parameter that has the best correlation with in vivo tumorigenicity (Freedman and Shin, 1974; Shin et al, 1975; Jeha et al, 1996), we next subjected all the cell clones to the analysis of clonogenic growth in soft agar. The most inhibited antisense clone (As2) had almost no ability of forming colonies in soft agar and intermediate phenotype was observed in the clone expressing intermediate level of PDPK $F_A$ (As1) as shown in Figure 2 and summarized in Table 1. The results demonstrate that specific suppression of PDPK $F_A$ expression is sufficient to inhibit proliferation and to diminish clonogenic potential of K562 cells in soft agar. Moreover, since non-transformed cells have a greater degree of dependence on serum for their growth than their transformed counterparts (Holley and Kiernan, 1968; Todaro and DeLarco, 1978; Jeha et al, 1996), we also determined the serum-dependent growth change in the antisense clones. K562 cells like most transformed cells, were able to grow well even in 0.5% serum (Figure 3). In sharp contrast, the antisense clone with low level of PDPK $F_A$ could not grow at all in 0.5% serum (Figure 3). Therefore, by reducing expression of PDPK $F_A$ in the antisense-transfected cells, a more normal phenotype, that of serum-dependent growth was restored. All the results taken together demonstrate that specific suppression of PDPK $F_A$ expression is sufficient to interfere with the growth properties of K562 cells.

### DISCUSSION

In this study, cloning of PDPK $F_A$ cDNA and its antisense expression vector and specific antibody were successfully established. The antisense expression vector could be stably introduced into human chronic myeloid leukaemia cells K562. Two stable antisense clones were subcloned, which constitutively suppressed ~30% and ~55% of the total PDPK $F_A$ activity in the untransfected

© 2000 Cancer Research Campaign 

parental cells respectively. Data presented here first demonstrate that endogenous PDPK $F_A$ expression in leukaemia cells can be suppressed by genetic engineering. Although the DNA sequence homology between PDPK $F_A$ and GSK-3β is ~85% (Woodgett, 1990), the constructed antisense vector presented here displayed little effect on the expression of GSK-3β in K562 cells. We also tested the effect of this PDPK $F_A$-specific antisense vector on the well-established PDPK member, namely Erk-1/-2, and again no suppression of Erk-1/-2 expression could be observed. The results taken together demonstrate that the antisense expression vector constructed here can potently and specifically suppress the endogenous PDPK $F_A$ expression in K562 cells.

By using the PDPK $F_A$-specific antisense expression vector developed in the present study, we further demonstrate that specific suppression of PDPK $F_A$ expression in the stable transfected antisense clones proportionally and potentially leads to a modification of the leukaemia malignant phenotypes. These included prolonged doubling time, poor clonogenic growth in soft agar and increased serum dependence. The decreased serum requirement of transformed cells has been attributed to autocrine growth stimulation by growth factor-like molecules that are produced by the cancer cells themselves (Holley and Kiernan, 1968; Todaro and DeLarco, 1978; Jeha et al, 1996). The increased serum requirement of antisense clones presented here provides evidence that PDPK $F_A$ may play an important role in autocrine growth stimulation process of chronic myeloid leukaemia. Moreover, since clonogenic growth in soft agar was established to have the best correlation with in vivo tumorigenicity (Freedman and Shin, 1974; Shin et al, 1975; Jeha et al, 1996), the diminished clonogenicity in antisense clones presented here strongly implicates that PDPK $F_A$ may play an essential role in regulating human chronic myeloid leukaemia cell growth. PDPK $F_A$ may, therefore, represent a potential new target for anticancer research. Whether suppression of PDPK $F_A$ activity using PDPK $F_A$ blockers such as genistein, kaempferol, apigenin and quercetin (Yang et al, 1998; Lee et al, 1998) may provide a useful clinic target for therapeutic intervention inhibiting the malignant behaviour of chronic myeloid leukaemia obviously presents an intriguing issue deserving further investigation. Nevertheless, the present study clearly demonstrates that specific suppression of proline-directed directed protein kinase $F_A$ is sufficient to interfere with the growth of human chronic myeloid leukaemia cells, providing an initial evidence for a critical role of this PDPK in leukaemia cell growth.

### ACKNOWLEDGEMENTS

This work was supported by a grant (NSC 89-2311-B-007-031) from the National Science Council of Taiwan, ROC. CCY is a postdoctoral fellow supported by NSC in Taiwan (NSC 89-2311-B-007-031). CPH and SFH are PhD students at the Department of Life Science, National Tsing Hua University in Taiwan.

### REFERENCES

Freedman VH and Shin SI (1974) Cellular tumorigenicity in nude mice: correlation with cell growth in semid solid medium. *Cell* **3**: 355–359

Gillespie PG and Hudspeth AJ (1991) Chemiluminescence detection of proteins from single cells. *Proc Natl Acad Sci USA* **88**: 2563–2567

He X, Saint-Jeannet JP, Woodgett JR, Varmus HE and Dawid IB (1995) Glycogen synthase kinase-3 and dorsoventral patterning in Xenopus embryos. *Nature* **374**: 617–622

Hemmings BA, Yellowlees D, Kernohan JC and Cohen P (1981) Purification of glycogen synthase kinase 3 from rabbit skeletal muscle. Copurification with the activating factor ($F_A$) of the (Mg-ATP) dependent protein phosphatase. *Eur J Biochem* **119**: 443–451

Holley RW and Kiernan JA (1968) 'Contact inhibition' of cell division in 3T3 cells. *Proc Natl Acad Sci USA* **60**: 300–304

Jeha S, Luo XN, Beran M, Kantarjian H and Atweh GF (1996) Antisense RNA inhibition of phosphoprotein p18 expression abrogates the transformed phenotype of leukemic cells. *Cancer Res* **56**: 1445–1450

Lee SC, Kuan CY, Yang CC and Yang SD (1998) Bioflavonoids commonly and potently induce tyrosine dephosphorylation/inactivation of oncogenic proline-directed protein kinase $F_A$ in human prostate carcinoma cells. *Anticancer Res* **18**: 1117–1122

Lee TT, Ho YS, Yu JS and Yang SD (1995) Overexpression of cellular activity and protein level of protein kinase $F_A$/GSK-3α correlates with human thyroid tumor cell dedifferentiation. *J Cell Biochem* **58**: 1–7

Lozzio CB and Lozzio BB (1975) Human chronic myelogenous leukemia cell-line with positive Philadelphia chromosome. *Blood* **45**: 321–334

Plyte SE, Hughes K, Nikolakaki E, Pulverer BJ and Woodgett JR (1992) Glycogen synthase kinase-3: functions in oncogenesis and development. *Biochem Biophys Acta* **1114**: 147–162

Reichlin M (1980) Use of glutaraldehyde as a coupling reagent for proteins and peptides. *Methods Enzymol* **70**: 159–165

Shin SI, Freedman VH, Risser R and Pollack R (1975) Tumorigenicity of virus-transformed cells in nude mice is correlated specifically with anchorage independenct growth in vitro. *Proc Natl Acad Sci USA* **72**: 4435–4439

Todaro GJ and DeLarco JE (1978) Growth factors produced by sarcoma virus-transformed cells. *Cancer Res* **38**: 4147–4154

Vandenheede JR, Yang SD, Goris J and Merlevede W (1980) ATP.Mg-dependent protein phosphatase from rabbit muscle. Purification of the activating factor and its regulation as a bifunctional protein also displaying synthase kinase activity. *J Biol Chem* **255**: 11768–11774

Woodgett JR (1990) Molecular cloning and expression of glycogen synthase kinase-3/Factor A. *EMBO J* **9**: 2431–2438

Yang CC, Hsu CP, Sheu JC, Mai XY and Yang SD (1998) Differential tyrosine phosphorylation/activation of oncogenic proline-directed protein kinase $F_A$/GSK-3α in well- and poorly-differentiated human prostate carcinoma cells. *J Protein Chem* **17**: 329–335

Yang SD (1994) Signal transduction and biological functions of protein phosphatase activating factor (protein kinase $F_A$/GSK-3α). *J Chin Biochem Society* **23**: 123–134

Yang SD, Yu JS and Wen ZD (1994) Tumor promoter phorbol ester reversibly modulates tyrosine dephosphorylation/inactivation of protein kinase $F_A$/GSK-3α in A431 cells. *J Cell Biochem* **56**: 550–558

Yang SD, Yu JS, Lee TT, Ni MH, Yang CC, Ho YS and Tsen TZ (1995) Association of protein kinase $F_A$/GSK-3α (a proline-directed kinase and a regulator of protooncogenes) with human cervical carcinoma dedifferentiation/progression. *J Cell Biochem* **59**: 143–150

Yang SD, Yu JS, Yang CC, Lee SC, Lee TT, Ni MH, Kuan CY and Chen HC (1996) Overexpression of protein kinase $F_A$/GSK-3α (a proline-directed protein kinase) correlates with human hepatoma dedifferentiation/progression. *J Cell Biochem* **61**: 238–245

Yu JS and Yang SD (1993) Immunological and biochemical study on tissue and subcellular distributions of protein kinase $F_A$ (an activating factor of ATP.Mg-dependent protein phosphatase). A simplified and efficient procedure for high quantity purification from brain. *J Protein Chem* **12**: 665–674

Yu JS and Yang SD (1994) Tyrosine dephosphorylation and inactivation of protein kinase $F_A$/GSK-3α by genistein in A431 cells. *J Cell Biochem* **56**: 131–141

 *© 2000 Cancer Research Campaign*
